# The Association Between Hyponatremia and Optic Nerve Sheath Diameter: A Prospective Study

**DOI:** 10.7759/cureus.34084

**Published:** 2023-01-23

**Authors:** Murat Duyan, Nafis Vural

**Affiliations:** 1 Emergency Medicine, Antalya Training and Research Hospital, Antalya, TUR; 2 Emergency Medicine, Akdeniz University Hospital, Antalya, TUR

**Keywords:** brain edema, emergency department, ocular ultrasound, optic nerve sheath diameter, hyponatremia

## Abstract

Background

Hyponatremia is a common electrolyte balance disorder. It may result in brain edema and increased intracranial pressure (ICP). Optic nerve sheath diameter (ONSD) measurement remains an increasingly sought-after method in many situations associated with ICP elevations. The aim of our study was to investigate the relationship between the change of ONSD before and after hypertonic saline (3% sodium chloride) treatment and the clinical improvement with increased sodium levels in patients with symptomatic hyponatremia who presented to the emergency department.

Methodology

This study was conducted in the emergency department of a tertiary hospital, according to the design of a prospective, self-controlled, non-randomized trial study. Determined by power analysis, 60 patients were included in the study. The statistical analysis of the continuous data was performed using the means, standard deviations, and minimum and maximum values of the feature values. The frequency and percentage values were used to define categorical variables. The mean difference comparison of pre-and post-treatment measurements was evaluated by paired t-test. P<0.05 was considered to be significant.

Results

The measurement parameters' differences before and after hypertonic saline treatment were evaluated. While the mean of the right eye ONSD was 5.27±0.22 mm before treatment, it declined substantially to 4.52±0.24 mm after treatment (p<0.001). It was also found that the left eye ONSD was 5.26±0.23 mm before the treatment and declined to 4.53±0.24 mm after the treatment (p<0.001). In addition, the mean of the overall ONSD was 5.26±0.23 mm before treatment and 4.52±0.24 mm after treatment (p<0.001).

Conclusions

Ultrasonic measurement of ONSD can be used to monitor the clinical improvement of patients receiving hypertonic saline therapy for symptomatic hyponatremia.

## Introduction

Hyponatremia is a common electrolyte balance disorder [1]. The European clinical practice guidelines define hyponatremia as a serum sodium (Na) level below 135 mmol/L [2]. The prevalence of hyponatremia in patients presenting to the emergency department (ED) ranges from 3% to 10%, depending on the environment and demographics of the local population [3,4]. Hyponatremia is primarily a water balance disorder in which body water is relatively high compared to total body sodium and potassium content [2].

Symptoms associated with hyponatremia range from mild nonspecific symptoms (dizziness, headache, or nausea) to severe life-threatening cerebral edema [5]. Due to the effective osmolality difference between the brain and plasma, brain cells begin to swell when water moves from the extracellular compartment to the intracellular compartment. This usually occurs when hyponatremia develops rapidly and the brain has little time to adapt to its hypotonic environment [2]. Eventually, cerebral edema causes an increase in intracranial pressure (ICP).

ICP is traditionally measured using invasive procedures such as lumbar puncture and ventriculostomy [6]. The measurement of optic nerve sheath diameter (ONSD) has received special attention among non-invasive methods. The optic nerve sheath (ONS) is a continuation of the dura mater and contains the subarachnoid space [7]. Thus, an increased ICP can be transmitted directly to the ONS through the subarachnoid space [6]. A correlation between ONSD and direct measurement of ICP has recently been demonstrated [8]. Bedside ultrasonography provides a simple, rapid, and indirect assessment of ICP by noninvasively measuring ONSD [9-11]. Using a linear probe that is carefully placed on the closed upper eyelid without applying pressure to the eye while the patient is in the supine position, ONSD is measured 3 mm behind the eyeball in two-dimensional mode [12].

ONSD measurement remains an increasingly sought-after method in many situations associated with ICP elevations. In a previous study, a significant difference in ONSD was observed after treatment compared to before in patients with hyponatremia [13]. However, there are very few studies in the literature on this subject.

The aim of our study was to investigate the relationship between the change of ONSD before and after hypertonic saline (3% sodium chloride) treatment and the clinical improvement with increased Na levels in patients with symptomatic hyponatremia who presented to the ED.

## Materials and methods

Study design and settings

This study was conducted in the emergency department of a tertiary hospital, according to the design of a prospective, self-controlled, non-randomized trial study. The Antalya Training and Research Hospital's Ethics Board approved the study (No. 2019-388, Decision No. 1/13, January 9, 2020). The study was carried out in accordance with the Helsinki Declaration.

Power analysis

The difference between ONSD measurement values before and after hypertonic saline treatment in patients with symptomatic hyponatremia was assessed using a quasi-experimental research design, and the effect size between measurements was determined to be 0.2 (minimum clinical significance). The number of patients enrolled in the study was determined as 60, with a maximum of 5% type 1 error and a minimum of 80% power.

Participants

The inclusion criteria were patients with symptomatic hyponatremia (Na <135 mmol/L) aged over 18 years. The exclusion criteria were age under 18 years, referrals from other facilities, facial injury influencing eyeballs, a previous eye condition that affects the optic nerve and/or orbital cavity, and clinical reasons for ICP increases (e.g., endocrinopathies, metabolic derangements, infectious and immunologic disorders, trauma, and malignancy). In addition, patients with poor-quality ultrasonographic measurements and inconsistent data were also excluded from the study. Seven patients were omitted from the study based on the exclusion criteria.

Study protocol

Sixty patients with symptomatic hyponatremia who presented to the ED were included in the study. Written informed consent was obtained from all patients or their first-degree relatives before participating in the study. Demographic characteristics (such as age and gender), vital signs, serum Na level, and ONSD measurement (before and after hypertonic saline treatment) were recorded.

Measurement of the optic nerve sheath diameter

**Figure 1:**
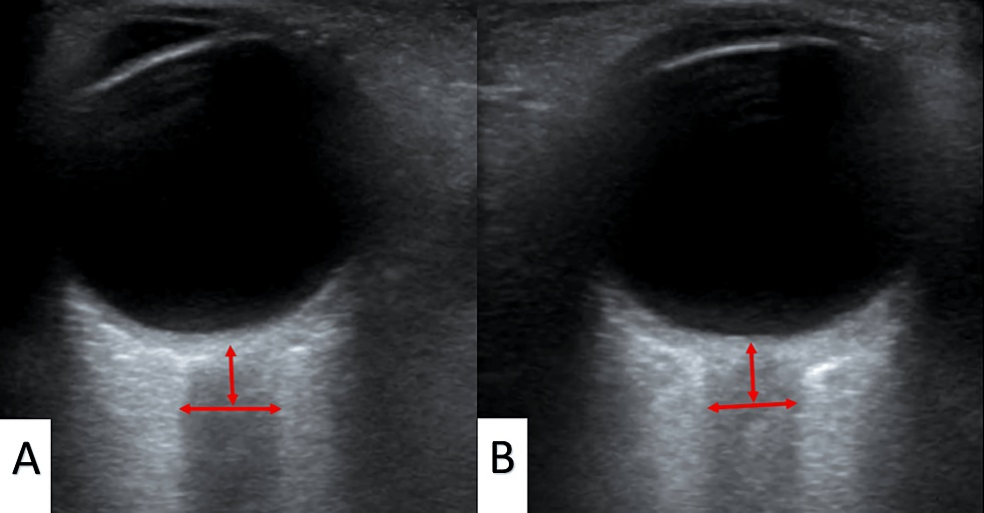
The optic nerve sheath diameter (ONSD) quantification technique before (A) and after (B) hypertonic saline treatment.

ONSD measurements were made with Mindray (Model DC-T6, Shenzhen, China) ultrasound in ocular imaging mode using 11 and 14 MHz linear transducers. The patients were instructed to wait for one minute in a semi-upright head and torso location (20°-30°) to avoid putting pressure on their eyes and to look forward with their eyes closed to prevent eye movement. The eyeball was filled with sterile gel, and measurements were made gently, without pressure on the eyeball, allowing a midline view over the closed eye. The ONSD was measured transversely from both eyes. The transverse diameter was measured 3 mm below the globe inlet after the screen was frozen at the location where the optic nerve entering the globe was most clearly visible (Figure 1). 

ONSD was assessed by emergency clinicians with at least two years of experience and who had accomplished a certified ultrasound course. The ultrasound images were transferred to the computer system. The images were interpreted by two trained and impartial radiologists. 

Hyponatremia management

Moderate symptoms include nausea, vomiting, confusion, and headache; and severe symptoms include vomiting, cardiorespiratory distress, abnormal and profound somnolence or coma, and seizures [2]. According to the European clinical practice guidelines, our patients with acute hyponatremia (onset <48 hours) with severe or moderate symptoms were promptly treated with 150 ml of hypertonic saline over 20 minutes, followed by a repeat measurement of the serum Na level 20 minutes later [2]. In patients with symptomatic hyponatremia in whom hypertonic saline administration may be repeated, the treatment goal was a rapid increase in serum Na of 5 mmol/L. The total serum Na level correction over 24 hours was a maximum of 8-10 mmol/L [2].

Data analysis

Parametric tests were used due to compliance with the central limit theorem [14]. The statistical analysis of the continuous data was performed using the means, standard deviations, and minimum and maximum values of the feature values. The frequency and percentage values were used to define categorical variables. The mean difference comparison of pre-and post-treatment measurements was evaluated by a paired t-test. The Pearson correlation coefficient was used to evaluate the relation between the continuous measurements. The coefficient of goodness of fit is given for the fit between the measurement methods. P<0.05 was considered to be significant. MedCalc statistical package software and New York software (e-Picos, New York, NY, USA, www.e-picos.com) were used to assess the data and ascertain the number of patients to be included in the study.

## Results

Of the study's 60 patients with symptomatic hyponatremia, 38 (63.3%) were male (Table 1).

**Table 1:** Distribution of sex characteristics.

Features	Groups	Number (n)	Percentage (%)
Sex	Female	22	36.7
	Male	38	63.3

The mean age of the patients was 59.93±5.3, and the age range was a minimum of 47 and a maximum of 71. Averages of the patient's vital signs were as follows: systolic blood pressure was 136.67±18.63 mmHg, diastolic blood pressure was 80.58±11.8 mmHg, pulse was 92.45±21.31 beats per minute, respiratory rate was 13.58±1.44 breaths per minute, and peripheral oxygen saturation was 96.24±1.47%. The median Na level of the patients before treatment was 121 mmol/L (minimum 105 mmol/L; maximum 126 mmol/L; and mean 119.9±5.45 mmol/L). The median Na level of the patients after hypertonic saline treatment was 124 mmol/L (minimum 111 mmol/L; maximum 129 mmol/L; and mean 123.77±4.68 mmol/L) (Table 2).

**Table 2:** Distribution of clinical characteristics. Min: minimum; Max: maximum; SD: standard deviation; SpO_2_: oxygen saturation; Na: sodium.

Features	Min-max	Median	Mean±SD
Age	47-71	59.9	59.93±5.3
Systolic blood pressure (mmHg)	90-160	140	136.67±18.63
Diastolic blood pressure (mmHg)	55-100	85	80.58±11.8
Pulse (minute)	60-125	85	92.45±21.31
Respiratory rate (minute)	12-16	14	13.58±1.44
SpO_2_ (%)	94-98	96	96.28±1.47
Pre-treatment Na (mmol/L)	105-126	121	119.9±5.45
Post-treatment Na (mmol/L)	111-129	124	123.77±4.68

**Figure 2:**
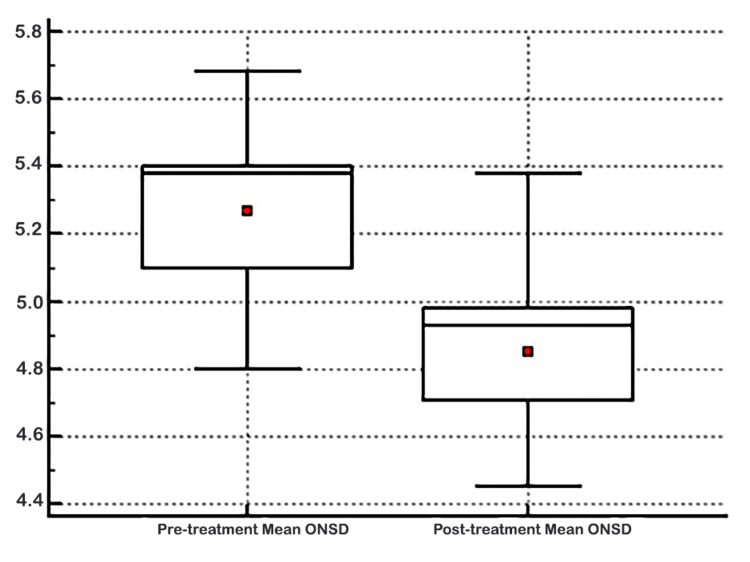
Evaluation of the difference in optic nerve sheath diameter (ONSD) before and after the treatment.

The measurement parameters' difference before and after hypertonic saline treatment was evaluated (Table 3, Figure 2). While the mean of the right eye ONSD was 5.27±0.22 mm before treatment, it declined substantially to 4.52±0.24 mm after treatment (p<0.001). It was also found that the left eye ONSD was 5.26±0.23 mm before the treatment and declined to 4.53±0.24 mm after the treatment (p<0.001). In addition, the mean of the overall ONSD was 5.26±0.23 mm before treatment and 4.52±0.24 mm after treatment (p<0.001).

**Table 3:** Evaluation of the difference in ONSD before and after the treatment. Paired t-test (P<0.05 significance). ONSD: optic nerve sheath diameter; SD: standard deviation.

	Pre-treatment		Post-treatment		P-value
	Median	Mean±SD	Median	Mean±SD	
ONSD right	5.38	5.27±0.22	4.9	4.84±0.23	<0.001
ONSD left	5.35	5.26±0.23	4.95	4.86±0.23	<0.001
The mean ONSD	5.37	5.26±0.23	4.93	4.85±0.23	<0.001

In evaluating the relationship between pre-treatment measurements, there is a low-grade negative significant correlation between the mean ONSD and Na (p=0.05). A low-grade correlation (r=−0.26) was also found between the mean ONSD and Na (Table 4). Moreover, after hypertonic saline treatment, there is a low-grade negative significant correlation between the mean ONSD and Na (p<0.05). A low negative correlation (r=−0.29) was found between the mean ONSD and Na (Table 4).

**Table 4:** Evaluation of the relationship and correlation between Na and ONSD. Significant at the level p<0.05 (Pearson correlation/goodness of fit coefficient). Na: sodium; ONSD: optic nerve sheath diameter.

		Na		
		Correlation coefficient	Goodness of fit coefficient	P-value
The mean ONSD	Pre-treatment	−0.26	0.05	0.05
	Post-treatment	−0.29	0.06	0.04

## Discussion

As hyponatremia deepens, neural damage due to brain edema increases [15]. The severity of hyponatremia and the intensity of the symptoms are significantly correlated [16]. Therefore, a diagnostic tool that can monitor serum Na status and ICP during the treatment process is essential for patients with symptomatic hyponatremia. Bedside ultrasonography is a diagnostic tool that has recently been used in many areas in the emergency department [17-19]. In this article, the importance of bedside ultrasonography in the management of patients with symptomatic hyponatremia in the ED was revealed.

ONSD normal ranges vary by race and population. On ultrasonographic measurement, the normal range of ONSD was 3.30-5.20 mm in Koreans, 2.65-4.30 mm in Chinese, and 2.4-4.7 mm in UK citizens [20-22]. Nevertheless, ONSD measurement was not performed in healthy adult patients in this study.

The direct relationship between increased ICP and ONSD in patients with traumatic and non-traumatic brain injuries has been demonstrated by previous studies [23,24]. Studies have suggested values greater than 0.55 cm for the diagnosis of ICP elevation but value greater than 0.6 cm have been found to correlate better with ICP elevation [25,26]. However, the normal ranges and critical values of ICP and ONSD to predict the prognosis of patients remains unclear. In the study of Naldi et al. in patients with cerebral hemorrhage, it was found that ONSD was significantly higher in patients with increased ICP (6.40±0.70 mm) than in patients without increased ICP (4.70±0.40 mm) [27]. Zhao et al. found the OSND to be 5.5±0.4mm in the death group and 4.4±0.5 mm in the survivors in their study of patients with stroke in the intensive care unit [28]. The reason for this may be the high amount of cerebral edema in patients with a mortal course. Chen et al. compared the ONSDs of patients who underwent lumbar punctures before and after the procedure. A sudden post-procedural decrease in ONSD was seen in 95% of patients, along with a reduction in ICP. Consequently, it was proved that ultrasonic measurement of ONSD can reflect relative real-time changes in ICP [29]. In this study, it was observed that ONSD measured after treatment in patients with symptomatic hyponatremia, which supports the literature, was significantly lower than before treatment. The reason for this may be that the water in the brain cells passes into the intravascular space thanks to the increased Na in the serum after the treatment, and the brain edema decreases.

According to the results of this study, a significant decrease in ONSD was observed after treatment in patients with symptomatic hyponatremia compared to before. ONSD measured by bedside ultrasonography, which is non-invasive, low-cost, reproducible, and fast, can be used in these patients during the course of treatment for ICP follow-up. In addition, using these patients as an alternative to Na monitoring during treatment can be recommended.

There are some limitations in this study. Hyponatremic patients were not divided into groups as hypovolemic, normovolemic, and hypervolemic. Since there was symptomatic hyponatremia, hypertonic saline treatment was started immediately after the ONSD measurement. The ages of our patients range from 47 to 71; therefore, these findings cannot be generalized and implemented across all age groups. This study could serve as a model for future controlled clinical trials.

## Conclusions

Hyponatremia is a life-threatening condition that increases ICP due to cerebral edema. There is a correlation between ONSD and ICP. During the treatment process of patients with symptomatic hyponatremia, the changes in ICP and serum Na can be indirectly monitored by ultrasonic measurement of ONSD. Therefore, ONSD measurement can be recommended in the clinical status follow-up of patients with symptomatic hyponatremia treated with hypertonic saline in the ED in terms of cost, practicality, reproducibility, and, most importantly, non-invasiveness.
